# AdvanTIG-206: a phase II, randomized study of ociperlimab plus tislelizumab and BAT1706 (bevacizumab biosimilar) versus tislelizumab and BAT1706 in first-line hepatocellular carcinoma

**DOI:** 10.1007/s00262-026-04399-8

**Published:** 2026-04-28

**Authors:** Zhenggang Ren, Yao Huang, Yabing Guo, Ming-Mo Hou, Wei Wang, Ming Kuang, Chunyi Hao, Wentao Wang, Yanqiao Zhang, Tianqiang Song, Chaoliu Dai, Hsing-Tao Kuo, Zinan Bao, Yunxia Zuo, Lei Wang, Fuxiang Zhu, Jia Fan

**Affiliations:** 1 Department of Hepatic Oncology, Liver Cancer Institute, Zhongshan Hospital, Fudan University, Shanghai, China; 2 Key Laboratory of Carcinogenesis and Cancer Invasion, Ministry of Education, Shanghai, China; 3 Mengchao Hepatobiliary Hospital of Fujian Medical University, Fuzhou, China; 4 Liver Tumor Center, Nanfang Hospital Southern Medical University, Guangzhou, China; 5 Division of Hematology and Oncology, Chang Gung Memorial Hospital, Taoyuan, Taiwan; 6 Department of Gastroenterology and Urology, Hunan Cancer Hospital, Changsha, China; 7 The First Affiliated Hospital, Sun Yat-Sen University, Guangzhou, China; 8 Beijing Cancer Hospital, Beijing, China; 9 West China Hospital Sichuan University, Chengdu, Sichuan China; 10 Gastroenterology Department, Harbin Medical University Cancer Hospital, Harbin, China; 11 Tianjin Medical University Cancer Institute & Hospital, Tianjin, China; 12 Shengjing Hospital of China Medical University, Shenyang, China; 13 Chi Mei Medical Center, Tainan, Taiwan; 14 School of Medicine, College of Medicine, National Sun Yat-sen University, Kaohsiung, Taiwan; 15 Solid Tumors, BeOne Medicines, Ltd, Shanghai, China; 16 Clinical Development, BeOne Medicines, Ltd, Shanghai, China; 17 Statistics, BeOne Medicines, Ltd, Beijing, China; 18 Clinical Biomarkers, BeOne Medicines, Ltd, Shanghai, China; 19 Department of Liver Surgery & Transplantation, Liver Cancer Institute, Zhongshan Hospital, Fudan University, Shanghai, China

**Keywords:** Hepatocellular carcinoma, Immune checkpoint inhibitor, Ociperlimab, Tislelizumab

## Abstract

**Background:**

Patients with hepatocellular carcinoma (HCC) have an unmet need for new therapies that improve survival. This phase II trial investigated the efficacy and safety of ociperlimab and tislelizumab plus BAT1706 (a bevacizumab biosimilar) in patients with first-line HCC.

**Methods:**

In this phase II, multicenter, randomized, multi-arm, open-label trial, patients with advanced HCC received ociperlimab and tislelizumab plus BAT1706 (Arm A) or tislelizumab plus BAT1706 (Arm B). The primary objective was to evaluate efficacy using objective response rate (ORR) assessed by the investigator per RESIST v1.1 for Arms A and B.

**Results:**

94 patients were randomized to Arm A (N = 62) and Arm B (N = 32). Confirmed ORR (95% confidence interval) was 37.1% (25.2–50.3) for Arm A and 40.6% (23.7–59.4) for Arm B. In Arms A and B, respectively, 90.3% and 80.6% of patients experienced treatment-related treatment-emergent adverse events (TEAEs), 59.7% and 32.3% experienced Grade ≥ 3 treatment-related TEAEs and 22.6% and 9.7% experienced treatment-related TEAEs leading to treatment discontinuation. Immune-mediated adverse events were reported in 50.0% of patients in Arm A and 45.2% of patients in Arm B. Infusion-related reactions occurred in a single patient in Arm A.

**Conclusion:**

In patients with advanced HCC, tislelizumab plus BAT1706 demonstrated promising ORR, while adding ociperlimab was not associated with improved efficacy. The safety profile of ociperlimab and tislelizumab plus BAT1706 was tolerable and manageable, with no new safety signals identified.

**Trial registration:**

ClinicalTrials.gov: NCT04948697 (September 20, 2021).

**Supplementary Information:**

The online version contains supplementary material available at 10.1007/s00262-026-04399-8.

## Introduction

Liver cancer is a major global health problem, with the World Health Organization reporting it as the sixth most common type of cancer in 2022, with 865,269 cases worldwide, and the third most common cause of cancer-related mortality, responsible for 757,948 deaths [1]. Hepatocellular carcinoma (HCC) is the most common form of liver cancer, accounting for approximately 90% of cases [2].

Sorafenib has been the standard-of-care for unresectable or metastatic HCC for almost two decades, despite it being difficult for patients to tolerate [3]. Recently, programmed death-ligand 1 (PD-L1) inhibitor plus bevacizumab, an anti-vascular endothelial growth factor (VEGF) inhibitor, has become the new standard-of-care for unresectable or metastatic HCC. Atezolizumab, a PD-L1 inhibitor, in combination with bevacizumab, is approved for the treatment of patients with unresectable or metastatic HCC who have not received prior systemic therapy based on results from the phase III IMBrave150 trial which reported a median overall survival (OS) (95% confidence interval [CI]) of 19.2 months (17.0–23.7) for atezolizumab plus bevacizumab compared with 13.4 months (11.4–16.9) for sorafenib, despite there being a higher rate of serious adverse events (AEs) associated with atezolizumab plus bevacizumab [4, 5]. Durvalumab, also a PD-L1 inhibitor, in combination with tremelimumab, is also approved for the treatment of patients with unresectable HCC, based on results from the phase III HIMALAYA trial, which reported a median OS (95% CI) of 16.4 months (14.2–19.6) for durvalumab plus tremelimumab, 16.6 months (14.1–19.1) for durvalumab, and 13.8 months (12.3–16.1) for sorafenib [6, 7]. Tislelizumab, a programmed cell death protein 1 (PD-1) inhibitor, is approved in China as a first-line treatment for unresectable or metastatic HCC, based on meeting the primary endpoint of OS non-inferiority in the phase III RATIONALE-301 trial, in which a median OS (95% CI) of 15.9 months (13.2–19.7) for tislelizumab and 14.1 (12.6–17.4) for sorafenib were reported [8, 9].

A potentially important step in the treatment of patients with HCC may be altering the tumor microenvironment to enhance the efficacy of immune checkpoint inhibitors [10]. Immune cells, including CD8 + T cells and CD8 + tumor-infiltrating lymphocytes, co-express the immune checkpoint receptors T-cell immunoglobulin and immunoreceptor tyrosine-based inhibitory motif domain (TIGIT) and PD-1, which converge on CD226 and regulate T-cell proliferation and function [11, 12]. Thus, combined treatment with an anti-TIGIT may alter the tumor microenvironment and improve the efficacy of anti-PD-1 treatment. In a murine model of autochthonous liver cancer, dual inhibition of TIGIT and PD-1 in autochthonous liver cancer resulted in synergistic inhibition of liver cancer growth [13].

A number of anti-TIGIT and anti-PD-1 combinations are currently in clinical development; however, there are few studies focused on HCC. The phase Ib/II Morpheus-Liver trial reported an objective response rate (ORR) (95% CI) of 42.5% (27.0–59.1) for tiragolumab, an anti-TIGIT, and atezolizumab plus bevacizumab, and 11.1% (1.4–34.7) for atezolizumab plus bevacizumab [14]. Rilvegostomig, a TIGIT/PD-1 bispecific antibody [15, 16], and vibostolimab, an anti-TIGIT [17], are also currently in clinical development for the treatment of solid tumors, including HCC.

Ociperlimab is a humanized Fc intact immunoglobulin (Ig) G1 monoclonal antibody designed to target TIGIT with high specificity and affinity, with preclinical evidence demonstrating that ociperlimab elicits strong immune responses and potent anti-tumor efficacy through multiple mechanisms of action [18]. Tislelizumab is an anti-PD-1 monoclonal antibody that blocks the PD-1/PD-L1 immune checkpoint, resulting in T-cell activation. BAT1706 is a bevacizumab biosimilar that is a recombinant humanized IgG1 monoclonal antibody which binds to VEGF with high affinity and inhibits tumor angiogenesis and the growth of tumors. Dual inhibition of VEGF and PD-L1 has shown immunomodulatory effects in other tumor types, as suggested by preclinical and clinical studies [19, 20]. Considering the antitumor activity associated with immunotherapy in HCC, adding an anti-TIGIT to an anti-PD-(L)1 and anti-VEGF receptor regimen may provide clinical benefit in patients with HCC.

This manuscript reports efficacy and safety results from AdvanTIG-206, a phase II, multicenter, randomized, multi-arm, open-label study of ociperlimab and tislelizumab plus BAT1706 in patients with advanced HCC who had not received prior systemic anticancer treatment.

## Methods

### Patients

Inclusion criteria included patients aged ≥ 18 years with histologically confirmed HCC (either Barcelona Clinic Liver Cancer [BCLC] Stage C disease or BCLC Stage B disease not amenable to or has progressed after loco-regional therapy and not amenable to a curative approach); had tumor tissue available for evaluable PD-L1 expression and other exploratory biomarkers; had not received prior systemic therapy, had ≥ 1 measurable legion as per Response Evaluation Criteria in Solid Tumors version 1.1 (RECIST v1.1); Eastern Cooperative Oncology Group (ECOG) Performance Status (PS) ≤ 1; adequate organ function determined ≤ 7 days prior to randomization as indicated by patients not requiring blood transfusion or growth factor support ≤ 14 days prior to screening for absolute neutrophil count ≥ 1.5 × 10^9^/L, platelets ≥ 75 × 10^9^/L or hemoglobin ≥ 90 g/L, an estimated glomerular filtration rate ≥ 30 mL/min/1.73 m^2^, serum albumin ≥ 29 g/L, serum total bilirubin ≤ 3 mg/dL, aspartate aminotransferase (AST) and alanine aminotransferase (ALT) ≤ 5 times upper limit of normal (ULN), proteinuria < 2 + (within 7 days of randomization), and an international normalized ratio or activated partial thromboplastin time ≤ 2 × ULN (for patients not receiving therapeutic anticoagulants); and females of childbearing potential must be willing to use a highly effective method of birth control for ≥ 120 days after the last dose of tislelizumab and have a negative urine or serum pregnancy test ≤ 7 days before randomization.

Exclusion criteria included patients with known fibrolamellar HCC, sarcomatoid HCC, and mixed cholangiocarcinoma and HCC histology; tumor thrombus involving the main trunk of the portal vein or inferior vena cava; prior therapy with an anti-PD-1, anti-PD-L1, anti-PD-L2, or any other antibody or drug specifically targeting T-cell co-stimulation or the checkpoint pathway; prior treatment with bevacizumab or a biosimilar; any major surgical procedure or any liver loco-regional therapy ≤ 28 days before randomization; any prior history of Grade ≥ 2 hepatic encephalopathy; active leptomeningeal disease or uncontrolled, untreated brain metastasis; active autoimmune diseases or history of autoimmune diseases that may relapse (excluding controlled type 1 diabetes, hypothyroidism, controlled celiac disease, skin diseases not requiring systemic treatment, and any other disease not expected to recur in the absence of external triggering factors); and any active malignancy ≤ 2 years before randomization except for the specific cancer under investigation.

### Trial design and interventions

**Fig. 1:**
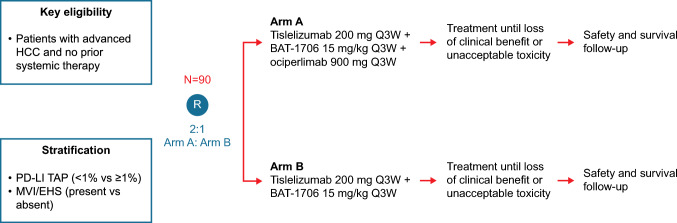
AdvanTIG-206 trial design. Abbreviations: EHS, extrahepatic spread; HCC, hepatocellular carcinoma; MVI, macrovascular invasion; PD-L1, programmed cell death protein–ligand 1; Q3W, every 3 weeks; R, randomization; TAP, tumor antigen positivity

This was a phase II, randomized, open-label, multicenter trial of ociperlimab plus tislelizumab and BAT1706 or tislelizumab and BAT1706 in first-line patients with advanced HCC conducted in Chinese mainland and Taiwan (ClinicalTrials.gov identifier: NCT04948697) (Fig. 1). Patients were randomly allocated in a 2:1 ratio to receive ociperlimab plus tislelizumab and BAT1706 (Arm A) or tislelizumab and BAT1706 (Arm B), respectively. Patients were randomized by permuted block stratified randomization with a stratification factor for PD-L1 expression (tumor area positivity [TAP] < 1% vs. ≥ 1%; TAP was formerly known as visually estimated combined positive score [vCPS]) and macrovascular invasion (MVI)/extrahepatic spread (EHS) (present vs. absent) by site personnel using an interactive response technology system.

Treatment was ociperlimab 900 mg intravenously (IV) every 3 weeks plus tislelizumab 200 mg IV every 3 weeks and BAT1706 15 mg/kg IV every 3 weeks (all over 21-day cycles), or tislelizumab 200 mg IV every 3 weeks and BAT1706 15 mg/kg IV every 3 weeks (all over 21-day cycles).

### Assessments

Tumor imaging was performed ≤ 28 days before randomization. During the trial, tumor imaging was performed approximately every 6 weeks (± 7 days) from Day 1 of Cycle 1 for the first 48 weeks, and every 12 weeks (± 7 days) thereafter, based on RECIST v1.1.

Safety was assessed by monitoring and recording all AEs. AEs were classified based on Medical Dictionary for Regulatory Activities version 26.0 (MedDRA v26.0) and graded according to the National Cancer Institute Common Terminology Criteria for Adverse Events version 5.0 (NCI-CTCAE v5.0). Laboratory values, vital signs, electrocardiograms, and physical examinations were used to assess safety. Treatment-emergent AEs (TEAEs) were any AE that had an onset date or a worsening in severity from baseline (pretreatment) on or after the first dose of study drug and up to 30 days after the last dose of study drug or initiation of new anticancer therapy, whichever occurred first. Immune-mediated AEs are of special interest in this trial and were assessed up to 90 days after last dose of study drug (regardless of initiation of new anticancer therapy), death, withdrawal of consent, or loss to follow-up, whichever occurred first. Recommendations for diagnostic evaluation and management of immune-mediated AEs are based on European Society for Medical Oncology and American Society of Clinical Oncology guidelines [21, 22].

Assessed biomarkers included PD-L1 TAP, which was centrally assessed using the VENTANA® PD-L1 (SP263) assay (Roche Diagnostics, Basel, Switzerland), and TIGIT expression on immune cells, which was centrally assessed by a formulation locked assay with the Roche SP410 assay.

### Outcomes

The primary objective was to evaluate the ORR, as determined by the investigator using RECIST v1.1, for Arms A and B**.**

Secondary objectives were to evaluate the duration of response (DOR), time to response (TTR), disease control rate (DCR), clinical benefit rate (CBR), progression-free survival (PFS), OS, and incidence and severity of TEAEs for Arms A and B.

Exploratory objectives were to potential biomarkers that may correlate with clinical responses/resistance to ociperlimab plus tislelizumab and BAT1706 and to tislelizumab and BAT1706 in Arms A and B, respectively.

### Statistical analysis

This trial was not designed to conduct formal hypothesis testing, but to obtain preliminary efficacy data; approximately 90 patients were planned to be enrolled with a 2:1 randomization ratio, with approximately 60 being allocated to the Arm A and 30 being allocated to Arm B.

The intention-to-treat (ITT) analysis set, which consisted of all randomized patients, was the primary analysis set for all efficacy analyses. The safety analysis set included all patients who received ≥ 1 dose of study drugs.

Best overall response was defined as the best response recorded from the randomization date until progressive disease or the initiation of new anticancer treatment, whichever occurred earlier.

Efficacy endpoints including ORR, DOR, TTR, DCR, CBR, PFS, and OS were summarized by arm in the ITT analysis set. ORR was defined as the proportion of patients with a confirmed complete response (CR) or partial response (PR) as assessed by the investigator, and was summarized using Clopper–Pearson 95% CIs to assess the precision of the point assessment. The Mantel–Haenszel common odds ratio (OR) was estimated along with its 95% CI constructed by a normal approximation of log OR and the Robins, Breslow, and Greenland variance estimate [23], stratified by PD-L1 expression and MVI/EHS. The *p*-value was obtained using the Cochran–Mantel–Haenszel method stratified by PD-L1 expression and MVI/EHS (*p*-value provided for descriptive purposes only). The Mantel–Haenszel common risk difference was estimated along with its 95% CIs constructed by a normal approximation and Sato’s variance estimator stratified by PD-L1 expression and MVI/EHS [24, 25].

DOR was defined as the time from the first confirmed objective response to disease progression after randomization or death (whichever occurred first). The median and other quartiles for DOR were estimated using the Kaplan–Meier methods and 2-sided 95% CIs were constructed with the Brookmeyer and Crowley method [26, 27]. DOR was censored at the last adequate tumor assessment if one of the following occurred: absence of event; the event occurred after a new anticancer therapy was given; or the event occurred after missing two or more consecutive tumor assessments. Clinical or symptomatic progressions without supporting radiologic data were not considered as events.

TTR was defined as the time from the randomization date to the first confirmed documented response. TTR was analyzed using descriptive statistics, such as mean, median, and standard deviation for patients who achieved an objective response.

DCR was defined as the proportion of patients who achieved confirmed CR, PR, or stable disease, and CBR was defined as the proportion of patients who achieved confirmed CR, PR, or durable stable disease (stable disease ≥ 24 weeks); both were analyzed using similar methods as described for ORR.

PFS was defined as the time from randomization to disease progression or death due to any cause (whichever occurred first), using similar method as described for DOR. Hazard ratios (HRs) and 95% CIs were estimated using a Cox regression model stratified by PD-L1 expression and MVI/EHS. The Efron method was used to handle ties if there were any. The *p*-value was calculated using a log-rank test stratified by PD-L1 expression and MVI/EHS (*p*-value provided for descriptive purposes only). The PFS censoring rule followed the same as that for DOR.

OS was analyzed using similar methods as described for PFS, except for censoring rules. For OS, patients were censored either at the date that the patient was last known to be alive, or at the date of data cutoff, whichever came earlier, in the absence of death.

Subgroup analyses of key efficacy endpoints were conducted to explore the consistency of efficacy across subgroups, including age, sex, ECOG PS, PD-L1 expression, MVI status, EHS status, viral status, BCLC stage at study entry, TIGIT expression and alpha-fetoprotein at baseline.

Missing dates or partially missing dates were imputed conservatively for AEs, disease history and prior therapy, prior/concomitant medications/procedures and subsequent anticancer therapy. For observed data analyses, missing data were not imputed, and only the observed records were included.

### Trial oversight

This trial was performed in accordance with the principles of the Declaration of Helsinki and Good Clinical Practice guidelines. The authors attest to the accuracy and completeness of the data and the fidelity of the trial to the protocol.

## Results

### Baseline characteristics

**Fig. 2:**
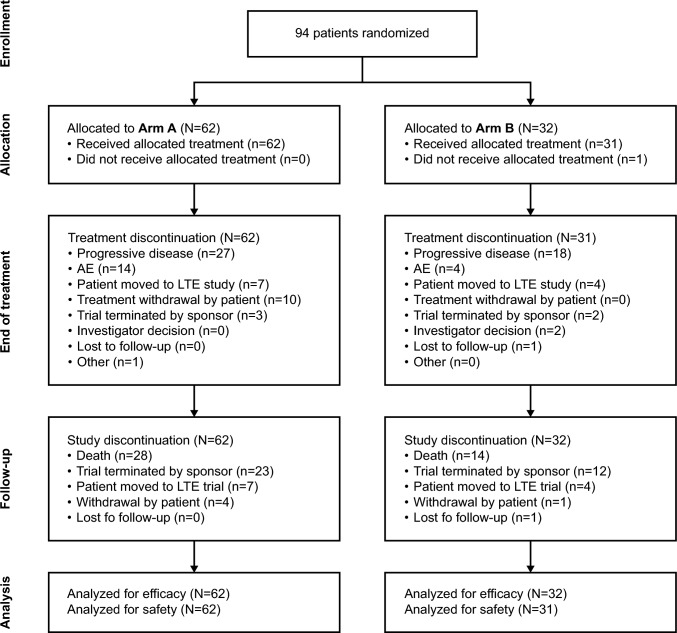
CONSORT diagram. Abbreviations: AE, adverse event; LTE, long-term extension

Between August 20, 2021 and the data cutoff date of February 1, 2024, a total of 94 patients were randomized to Arm A (N = 62) and Arm B (N = 32) (Fig. 2). The median study follow-up time (range) was 18.3 months (0.4–26.4 months) for Arm A and 18.1 months (0.1–26.9 months) for Arm B.

Baseline characteristics were generally comparable across both arms (Table 1).

**Table 1:** Demographics and baseline characteristics (ITT analysis set)

Characteristic	Arm A Ociperlimab + Tislelizumab + BAT1706 (N = 62)	Arm B Tislelizumab + BAT1706 (N = 32)	Total (N = 94)
Age, median (range), years	57.0 (28–85)	60.0 (30–78)	58.5 (28–85)
Male, n (%)	57 (91.9)	30 (93.8)	87 (92.6)
ECOG PS 1, n (%)	23 (37.1)	14 (43.8)	37 (39.4)
Tobacco use status, n (%)			
Never	27 (43.5)	14 (43.8)	41 (43.6)
Current	14 (22.6)	9 (28.1)	23 (24.5)
Former	21 (33.9)	9 (28.1)	30 (31.9)
Alcohol consumption status, n (%)			
Never	34 (54.8)	15 (46.9)	49 (52.1)
Current	5 (8.1)	2 (6.3)	7 (7.4)
Former	23 (37.1)	15 (46.9)	38 (40.4)
BCLC initial staging, n (%)			
Stage A	12 (19.4)	5 (15.6)	17 (18.1)
Stage B	22 (35.5)	12 (37.5)	34 (36.2)
Stage C	23 (37.1)	15 (46.9)	38 (40.4)
Missing	5 (8.1)	0 (0.0)	5 (5.3)
PD-L1 expression per IRT, n (%)			
< 1%	33 (53.2)	18 (56.3)	51 (54.3)
≥ 1%	29 (46.8)	14 (43.8)	43 (45.7)
TIGIT expression, n (%)*			
< 1%	28 (45.2)	16 (50.0)	44 (46.8)
≥ 1%	32 (51.6)	16 (50.0)	48 (51.1)
Not applicable	2 (3.2)	0 (0)	2 (2.1)
MVI/EHS per IRT, n (%)			
Present	41 (66.1)	21 (65.6)	62 (66.0)
Absent	21 (33.9)	11 (34.4)	32 (34.0)
Histological type at study entry, n (%)			
Well differentiated	4 (6.5)	1 (3.1)	5 (5.3)
Moderately differentiated	10 (16.1)	7 (21.9)	17 (18.1)
Poorly differentiated	6 (9.7)	6 (18.8)	12 (12.8)
Unknown	42 (67.7)	18 (56.3)	60 (63.8)
Viral status, n (%)			
HBV infected only	52 (83.9)	24 (75.0)	76 (80.9)
HCV infected only	2 (3.2)	3 (9.4)	5 (5.3)
HBV and HCV co-infected	1 (1.6)	0 (0.0)	1 (1.1)
Uninfected^†^	7 (11.3)	5 (15.6)	12 (12.8)
Other relevant medical history, n (%)	46 (74.2)	27 (84.4)	73 (77.7)
Alcoholic hepatitis	3 (4.8)	0 (0.0)	3 (3.2)
NASH/fatty liver	1 (1.6)	1 (3.1)	2 (2.1)
Underlying cirrhosis	39 (62.9)	20 (62.5)	59 (62.8)
Partial or complete portal vein thrombosis	13 (21.0)	7 (21.9)	20 (21.3)
Esophageal varices	11 (17.7)	12 (37.5)	23 (24.5)
Ascites	10 (16.1)	4 (12.5)	14 (14.9)
Jaundice	1 (1.6)	0 (0.0)	1 (1.1)
Alpha-fetoprotein at baseline (ng/mL), n (%)			
≤ 200	35 (56.5)	19 (59.4)	54 (57.4)
> 200–400	4 (6.5)	0 (0.0)	4 (4.3)
> 400	18 (29.0)	9 (28.1)	27 (28.7)
Missing	5 (8.1)	4 (12.5)	9 (9.6)

BCLC, Barcelona Clinical Liver Cancer; ECOG PS, Eastern Cooperative Oncology Group Performance Status; EHS, extrahepatic spread; HBV, hepatitis B virus; HCV, hepatitis C virus; IRT, interactive response technology; ITT, intention-to-treat; MVI, microvascular invasion; NASH, non-alcoholic steatohepatitis; PD-L1, programmed cell death protein–ligand 1; TIGIT, T-cell immunoglobulin and immunoreceptor tyrosine-based inhibitory motif domain

^*^TIGIT expression is available in 60 patients in Arm A and 32 patients in Arm B. Two patients in Arm A have no TIGIT expression results due to technical issues

^†^ “Uninfected” includes patients without a medical history of HBV and/or HCV infections

### Efficacy

Confirmed ORR (95% CI) as determined by the investigator was 37.1% (25.2–50.3) for Arm A and 40.6% (23.7–59.4) for Arm B, representing a risk difference of − 3.9% (95% CI − 25.5–17.7) (Table 2).

**Table 2:** Efficacy endpoints (ITT analysis set)

Characteristic	Arm A Ociperlimab + tislelizumab + BAT1706 (N = 62)	Arm B Tislelizumab + BAT1706 (N = 32)
Best overall response, n (%)		
CR	0 (0.0)	0 (0.0)
PR	23 (37.1)	13 (40.6)
SD	25 (40.3)	10 (31.3)
PD	10 (16.1)	7 (21.9)
Not evaluable	4 (6.5)	2 (6.3)
ORR, n (%)	23 (37.1)	13 (40.6)
95% CI*	25.2–50.3	23.7–59.4
OR (95% CI)^†^	0.86 (0.37–2.00)	–
Risk difference (95% CI), %^‡^	− 3.9 (− 25.5 to 17.7)	–
2-sided *p*-value^§^	0.7167	
Median DOR (95% CI), months^‖^	12.6 (6.9–NE)	12.4 (5.4–NE)
Median TTR (range), months	2.8 (1.2–6.9)	4.2 (1.4–8.3)
DCR, n (%)	48 (77.4)	23 (71.9)
95% CI*	65.0–87.1	53.3–86.3
CBR, n (%)	37 (59.7)	18 (56.3)
95% CI*	46.4–71.9	37.7–73.6
Median PFS (95% CI), months^‖^	8.3 (5.5–10.3)	6.9 (4.1–17.4)
Stratified HR (95% CI)^¶^	1.11 (0.66–1.88)	–
Median OS (95% CI), months^‖^	19.7 (18.4–NE)	22.9 (14.4–NE)
Stratified HR (95% CI)^¶^	1.01 (0.53–1.93)	–

CBR, clinical benefit rate; CI, confidence interval; CR, complete response; DCR, disease control rate; DOR, duration of response; EHS, extrahepatic spread; HR, hazard ratio; ITT, intention-to-treat; MVI, macrovascular invasion; NE, not estimable; OR, odds ratio; ORR, objective response rate; OS, overall survival; PD, progressive disease; PD-L1, programmed cell death protein–ligand 1; PFS, progression-free survival; PR, partial response; SD, stable disease; TTR, time to response

^*^The 95% CI was estimated using the Clopper–Pearson method

^†^Mantel–Haenszel common OR was estimated along with its 95% CI constructed by a normal approximation of log OR and the Robins, Breslow, and Greenland variance estimate stratified by PD-L1 expression and MVI/EHS

^‡^Mantel–Haenszel common risk difference was estimated along with its 95% CIs constructed by a normal approximation and Sato’s variance estimator stratified by PD-L1 expression and MVI/EHS

^§^The *p*-value was obtained using the Cochran–Mantel–Haenszel method stratified by PD-L1 expression and MVI/EHS. *p*-Value is for descriptive purpose only

^‖^Medians were estimated by the Kaplan–Meier method with 95% CIs estimated using the Brookmeyer and Crowley method with log–log transformation

^¶^HR and 95% CIs were estimated using a Cox regression model stratified by PD-L1 expression and MVI/EHS. Efron method were used to handle ties if there were any

**Fig. 3:**
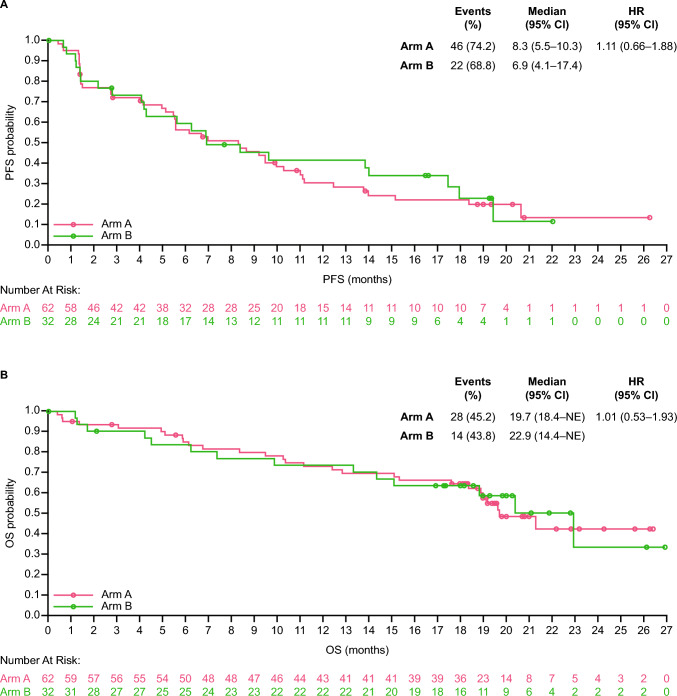
Kaplan–Meier curves for PFS (**A**) and OS (**B**). Abbreviations: NE, not estimable; OS, overall survival; PFS, progression-free survival

Median DOR (95% CI) was 12.6 months (6.9–not estimable [NE]) for Arm A and 12.4 months (5.4–NE) for Arm B. Median TTR (range) was 2.8 months (1.2–6.9) for Arm A and 4.2 months (1.4–8.3) for Arm B. DCR (95% CI) was 77.4% (65.0–87.1) for Arm A and 71.9% (53.3–86.3) for Arm B (Table 2). CBR (95% CI) was 59.7% (46.4–71.9) for Arm A and 56.3% (37.7–73.6) for Arm B (Table 2). Median PFS (95% CI) as determined by the investigator was 8.3 months (5.5–10.3) for Arm A and 6.9 months (4.1–17.4) for Arm B, with a stratified HR (95% CI) of 1.11 (0.66–1.88) (Table 2/Fig. 3A). Median OS (95% CI) was 19.7 months (18.4–NE) for Arm A and 22.9 months (14.4–NE) for Arm B, with a stratified HR of 1.01 (0.53–1.93) (Table 2/Fig. 3B).

Subgroup analysis of ORR showed that in patients with baseline PD-L1 expression of ≥ 1%, ORR was numerically higher in Arm A (48.3%) than Arm B (35.7%), while the opposite trend was observed in the PD-L1 < 1% subgroup (Supplementary Fig. S1). In patients with baseline TIGIT expression ≥ 1%, ORR was 37.5% in both Arms A and B; in patients with baseline TIGIT expression of < 1%, ORR was numerically higher in Arm B (43.8%) than in Arm A (32.1%) (Supplementary Fig. S1). Subgroup analysis of PFS and OS showed comparable results between Arm A and Arm B for PD-L1 expression < 1% and ≥ 1% and TIGIT expression < 1% and ≥ 1% (Supplementary Fig. S2/S3). However, subgroup analyses should be treated with caution due to the small sample sizes.

### Safety

Median duration of exposure (range) was 7.24 months (0.3–23.5 months) for ociperlimab and tislelizumab and 6.90 months (0.3–23.5 months) for BAT1706 in Arm A; median duration of exposure was 9.03 months (0.7–27.6 months) for tislelizumab and 9.03 months (0.7–23.5 months) for BAT1706 in Arm B (Supplementary Table S1).

All patients experienced at least one TEAE (Table 3). The three most common TEAEs were proteinuria, hypertension, and AST increased (Supplementary Table S2). The proportion of patients who experienced at least one treatment-related TEAE was numerically higher in Arm A (90.3% [56/62]) than Arm B (80.6% [25/31]). The four most common treatment-related TEAEs were proteinuria, hypertension, AST increased, and ALT increased (Supplementary Table S3). Grade ≥ 3 treatment-related TEAEs occurred in a higher percentage of patients in Arm A (59.7%) than Arm B (32.3%). The three most common Grade ≥ 3 treatment-related TEAEs were hypertension, proteinuria, and rash. The three most common tislelizumab/ociperlimab-related TEAEs were AST increased, ALT increased, and proteinuria (Supplementary Table S4).

**Table 3:** Overview of AEs (safety analysis set)

	Arm A Ociperlimab + tislelizumab + BAT1706 (N = 62) n (%)	Arm B Tislelizumab + BAT1706 (N = 31) n (%)
Patients with any TEAE	62 (100.0)	31 (100.0)
Grade ≥ 3	48 (77.4)	17 (54.8)
Serious	31 (50.0)	12 (38.7)
Leading to death*	3 (4.8)	0 (0.0)
Leading to treatment discontinuation	18 (29.0)	6 (19.4)
Patients with any treatment-related TEAE	56 (90.3)	25 (80.6)
Grade ≥ 3	37 (59.7)	10 (32.3)
Serious	19 (30.6)	4 (12.9)
Leading to death*^†^	3 (4.8)	0 (0.0)
Leading to treatment discontinuation	14 (22.6)	3 (9.7)
Patients with any treatment-related TEAE related to ociperlimab/tislelizumab	55 (88.7)	24 (77.4)
Grade ≥ 3	29 (46.8)	6 (19.4)
Serious	14 (22.6)	4 (12.9)
Leading to death*	2 (3.2)	0 (0.0)
Patients with any treatment-related TEAE related to BAT1706	54 (87.1)	25 (80.6)
Grade ≥ 3	31 (50.0)	8 (25.8)
Serious	10 (16.1)	3 (9.7)
Leading to death*	2 (3.2)	0 (0.0)
Patients with any immune-mediated AE	31 (50.0)	14 (45.2)
Patients with IRRs	1 (1.6)	0 (0.0)

AEs were classified based on MedDRA v26.0. AEs were graded for severity using CTCAE v5.0. TEAE is defined as an AE that had an onset date or a worsening in severity from baseline (pretreatment) on or after the first dose of study drug(s) up to 30 days following last dose of study drug(s) or initiation of a new anticancer therapy, whichever occurs first. Treatment-related TEAEs include those events considered by the investigator to be related or with missing assessment of the causal relationship

AE, adverse event; CTCAE v5.0, Common Terminology Criteria for Adverse Events version 5.0; IRR, infusion-related reaction; MedDRA v26.0, Medical Dictionary for Regulatory Activities version 26.0; TEAE, treatment-emergent adverse event

^*^The summary of TEAE leading to death only include TEAE leading to death excluding death due to disease under study

^†^Treatment-related TEAEs that led to death included upper gastrointestinal perforation (assessed as related to ociperlimab, tislelizumab, and BAT1706), cerebral ischemia (assessed as related to BAT1706), and hepatitis (assessed as related to tislelizumab)

Treatment-related serious TEAEs occurred in a higher percentage of patients in Arm A (30.6% [19/62]) than Arm B (12.9% [4/31]) (Table 3). Treatment-related TEAEs that led to treatment discontinuation were numerically higher in Arm A (22.6% [14/62]) than Arm B (9.7% [3/31]) (Table 3); all events were reported in a single patient each (except for rash, which occurred in two patients in Arm A). Treatment-related TEAEs that led to death, excluding death due to the disease under study, occurred in 4.8% (3/62) of patients in Arm A and no patients in Arm B. These fatal treatment-related TEAEs were upper gastrointestinal perforation (assessed as related to ociperlimab, tislelizumab, and BAT1706), cerebral ischemia (assessed as related to BAT1706), and hepatitis (assessed as related to tislelizumab) (Table 3).

Immune-mediated AEs occurred in a similar percentage of patients in Arms A (50.0% [31/62]) and Arm B (45.2% [14/31]). Immune-mediated AEs that had a ≥ 5% incidence increase in Arm A compared with Arm B were immune-mediated pneumonitis (6.5% [4/62] in Arm A vs. 0% [0/31] in Arm B) and immune-mediated skin adverse reactions (37.1% [23/62] in Arm A vs. 22.6% [7/31] in Arm B) (Supplementary Table S5). Grade ≥ 3 immune-mediated AEs occurred in a higher percentage of patients in Arm A (22.6% [14/62]) than Arm B (9.7% [3/31]) (Supplementary Table S6). Immune-mediated AEs leading to treatment discontinuation occurred in less than 10% of patients in Arms A (9.7% [6/62]) and B (3.2% [1/31]) (Supplementary Table S6). A single patient in Arm A and no patients in Arm B experienced an immune-mediated AE that led to death; the corresponding AE was immune-mediated hepatitis (Supplementary Table S6). A single patient in Arm A and no patients in Arm B experienced an infusion-related AE.

## Discussion

Recent clinical developments in the treatment of HCC have involved combining anti-PD-1/PD-L1 monoclonal antibodies with novel agents targeting lymphocyte-activation gene 3 (LAG-3), T-cell immunoglobulin mucin-3 (TIM-3) or TIGIT [28]. Preclinical studies suggest that combining an anti-TIGIT and anti-PD-1 monoclonal antibody may enhance anti-tumor activity of the latter [29].

As mentioned previously, several recent trials, including IMbrave150 [4], HIMALAYA [7], and Morpheus-Liver [14], have demonstrated the efficacy of immunotherapy in unresectable HCC. Additionally, recent results from the phase III CheckMate-9DW trial reported a significant improvement in efficacy for nivolumab plus ipilimumab compared with lenvatinib/sorafenib, with a higher ORR [95% CI] (36% [31–42] vs. 13% [10–17]) with durable responses, and median OS [95% CI] (23.7 months [18.8–29.4] vs. 20.6 months [17.5–22.5]) [30]. Of note, in AdvanTIG-206, the ORR in patients who received ociperlimab and tislelizumab plus BAT1706 or tislelizumab plus BAT1706 exceeded 36%; however, the addition of ociperlimab to tislelizumab plus BAT1706 did not provide additional improvement in ORR. Although trials of similar combinations, such as tiragolumab and atezolizumab plus bevacizumab in the MORPHEUS-Liver trial reported significant ORR increase as compared to atezolizumab plus bevacizumab, it is anticipated whether this can be confirmed and translated into statistically significant survival benefits in pivotal trials. Additionally, trials of TIGIT/PD-1 bispecific antibodies, such as rilvegostomig have been initiated [15, 16, 31], with the expectation of improved efficacy, lower toxicity, as well as convenience for patients. It should be noted that caution is advised if comparing the results between these trials with AdvanTIG-206 because of differences in the underlying patient population, for example differences in viral status.

In general, although adding ociperlimab to tislelizumab plus BAT1706 was not associated with an improvement in ORR compared with tislelizumab plus BAT1706, tislelizumab plus BAT1706 demonstrated promising ORR and extended median OS in patients with advanced HCC in AdvanTIG-206. In terms of secondary objectives, DOR, TTR, DCR, and CBR were generally similar in both trial arms, and OS and PFS did not show meaningful differences with sufficient follow-up. It should be noted that caution is required in data interpretation, as the overall patient sample size was small, thus potentially impacting the interpretation of the results, particularly subgroup analyses for efficacy by PD-L1 and TIGIT expression; the study was conducted exclusively in Mainland China and Taiwan and enrolled only Chinese patients; and the study was not designed for formal hypothesis testing. Future trials investigating anti-PD-1 and anti-TIGIT combinations should include multiracial populations globally, larger sample sizes, and formal hypothesis testing, to address concerns about this treatment regimen.

Overall, ociperlimab and tislelizumab plus BAT1706 and tislelizumab plus BAT1706 were tolerable, with no new safety signals identified beyond the known safety profiles of immune checkpoint inhibitors and VEGF inhibitors. A general increase in the TEAE incidence rate was observed for ociperlimab and tislelizumab plus BAT1706 compared with tislelizumab plus BAT1706; however, the most commonly reported TEAEs, including AST increased, ALT increased, platelet count decreased, pruritus, and hyperuricemia, were in line with the safety profiles of tislelizumab and BAT1706 and were consistent with the clinical expectation in this advanced HCC population. For tislelizumab/ociperlimab-related TEAEs, a higher incidence of events with an incidence difference ≥ 10% was observed for ociperlimab and tislelizumab plus BAT1706 compared with tislelizumab plus BAT1706; however, most were grade 1 or 2 events. Grade ≥ 3 tislelizumab/ociperlimab-related TEAEs with a ≥ 2% incidence difference for ociperlimab and tislelizumab plus BAT1706 compared with tislelizumab plus BAT1706 were AST increased (3.2% vs. 0.0%), rash (9.7% vs. 0.0%), and proteinuria (8.1% vs. 3.2%).

## Conclusions

In the phase II AdvanTIG-206 trial, tislelizumab plus BAT1706 demonstrated promising ORR, while adding ociperlimab to tislelizumab plus BAT1706 was not associated with improved ORR, PFS, or OS. The safety profiles of ociperlimab and tislelizumab plus BAT1706 and tislelizumab plus BAT1706 were tolerable. Further investigation of new immunotherapy agents or combination strategies is encouraged to improve the efficacy of first-line treatment of HCC.

## Supplementary Information

Below is the link to the electronic supplementary material.

Supplementary file1 (PDF 1154 KB)

## Data Availability

On request, and subject to certain criteria, conditions, and exceptions, BeOne Medicines, Ltd. will provide access to individual deidentified participant data from applicable BeOne Medicines-sponsored studies. BeOne Medicines shares data only when permitted by applicable data privacy and security laws and regulations, shares when it is feasible to do so without compromising the privacy of the study participants, and other considerations. Data requests may be submitted to ClinicalTrials@beonemed.com.
