# Supplementary material for: AdvanTIG-206: a phase II, randomized study of ociperlimab plus tislelizumab and BAT1706 (bevacizumab biosimilar) versus tislelizumab and BAT1706 in first-line hepatocellular carcinoma
Source: Cancer Immunol Immunother. 2026 Apr 28;75(5):158. doi: 10.1007/s00262-026-04399-8 (PMC13125556; doi:10.1007/s00262-026-04399-8)
Supplement: Supplementary file 1 — Supplementary file1 (PDF 1154 KB) [file 262_2026_4399_MOESM1_ESM.pdf]

Supplement

AdvanTIG-206: a phase II, randomized study of ociperlimab plus tislelizumab and BAT1706 (bevacizumab biosimilar) versus tislelizumab and BAT1706 in first-line hepatocellular carcinoma

Cancer Immunology, Immunotherapy (submitted in 2026) - Zhenggang Ren et al

TABLES

Supplementary Table S1. Duration of exposure (safety analysis set)

	**Arm A Ociperlimab + tislelizumab + BAT1706 (N = 62)**			**Arm B Tislelizumab + BAT1706 (N = 31)**	
	**Ociperlimab**	**Tislelizumab**	**BAT1706**	**Tislelizumab**	**BAT1706**
Median duration (range), months*	7.24 (0.3–23.5)	7.24 (0.3–23.5)	6.90 (0.3–23.5)	9.03 (0.7–27.6)	9.03 (0.7–23.5)

*Duration of exposure (months) of ociperlimab, tislelizumab, and BAT1706 are calculated as (last date of exposure − first dose date + 1)/30.4375, where data cutoff date is used as last date of exposure for ongoing patients, and minimum (cutoff date, death date, last dose date + 20) is used for discontinued patients (with non-missing end of treatment date).

Supplementary Table S2. TEAEs in ≥10% of patients (safety analysis set)

**Preferred Term**	**Arm A Ociperlimab + tislelizumab + BAT1706 (N = 62) n (%)**		**Arm B Tislelizumab + BAT1706 (N = 31) n (%)**	
	**Any grade**	**Grade ≥3**	**Any grade**	**Grade ≥3**
Patients with any TEAE	62 (100.0)	48 (77.4)	31 (100.0)	17 (54.8)
Aspartate aminotransferase increased	26 (41.9)	3 (4.8)	9 (29.0)	2 (6.5)
Proteinuria	24 (38.7)	7 (11.3)	16 (51.6)	2 (6.5)
Alanine aminotransferase increased	23 (37.1)	1 (1.6)	8 (25.8)	1 (3.2)
Hypertension	23 (37.1)	12 (19.4)	15 (48.4)	5 (16.1)
Platelet count decreased	23 (37.1)	1 (1.6)	8 (25.8)	3 (9.7)
Rash	16 (25.8)	6 (9.7)	6 (19.4)	0 (0.0)
Anemia	13 (21.0)	2 (3.2)	4 (12.9)	2 (6.5)
Pruritus	13 (21.0)	0 (0.0)	2 (6.5)	0 (0.0)
Pyrexia	13 (21.0)	0 (0.0)	4 (12.9)	0 (0.0)
COVID-19	12 (19.4)	1 (1.6)	6 (19.4)	0 (0.0)
Hypoalbuminemia	12 (19.4)	0 (0.0)	10 (32.3)	0 (0.0)
Hyperuricemia	11 (17.7)	0 (0.0)	2 (6.5)	0 (0.0)
Blood alkaline phosphatase increased	10 (16.1)	1 (1.6)	1 (3.2)	1 (3.2)
Blood bilirubin increased	10 (16.1)	1 (1.6)	8 (25.8)	2 (6.5)
Diarrhea	10 (16.1)	0 (0.0)	10 (32.3)	0 (0.0)
Hypokalemia	10 (16.1)	2 (3.2)	4 (12.9)	3 (9.7)
Decreased appetite	9 (14.5)	0 (0.0)	5 (16.1)	0 (0.0)
White blood cell count decreased	9 (14.5)	0 (0.0)	3 (9.7)	0 (0.0)
Fatigue	8 (12.9)	0 (0.0)	3 (9.7)	0 (0.0)
Hyponatremia	8 (12.9)	3 (4.8)	3 (9.7)	0 (0.0)
Weight decreased	8 (12.9)	1 (1.6)	4 (12.9)	0 (0.0)

Blood creatine phosphokinase increased	7 (11.3)	0 (0.0)	2 (6.5)	0 (0.0)
Constipation	7 (11.3)	0 (0.0)	2 (6.5)	0 (0.0)
Dizziness	7 (11.3)	0 (0.0)	1 (3.2)	0 (0.0)
Gamma-glutamyltransferase increased	7 (11.3)	1 (1.6)	2 (6.5)	1 (3.2)
Hypothyroidism	7 (11.3)	0 (0.0)	9 (29.0)	1 (3.2)

AEs were classified based on MedDRA version 26.0. AEs were graded for severity using CTCAE v5.0. Patients with multiple events for a given Preferred Term were counted once at the Preferred Term level. Events were sorted by decreasing frequency of Preferred Term in 'Any Grade' of Arm A. Events were cut per Preferred Term ≥10% of 'Any Grade' of Arm A.

Abbreviations: AE, adverse event; CTCAE v5.0, Common Terminology Criteria for Adverse Events version 5.0; TEAE, treatment-emergent adverse event.

Supplementary Table S3. Treatment-related TEAEs in ≥5% of patients (safety analysis set)

Preferred Term	Arm A<br>Ociperlimab + tislelizumab + BAT1706<br>(N = 62)<br>n (%)		Arm B<br>Tislelizumab + BAT1706<br>(N = 31)<br>n (%)	
	**Any grade**	**Grade ≥3**	**Any grade**	**Grade ≥3**
Patients with at least one treatment-related TEAE	56 (90.3)	37 (59.7)	25 (80.6)	10 (32.3)
Proteinuria	23 (37.1)	7 (11.3)	16 (51.6)	2 (6.5)
Aspartate aminotransferase increased	21 (33.9)	2 (3.2)	5 (16.1)	0 (0.0)
Alanine aminotransferase increased	20 (32.3)	1 (1.6)	5 (16.1)	0 (0.0)
Hypertension	18 (29.0)	11 (17.7)	10 (32.3)	5 (16.1)
Platelet count decreased	18 (29.0)	0 (0.0)	6 (19.4)	3 (9.7)
Rash	13 (21.0)	6 (9.7)	5 (16.1)	0 (0.0)
Pruritus	9 (14.5)	0 (0.0)	2 (6.5)	0 (0.0)
Blood bilirubin increased	8 (12.9)	1 (1.6)	6 (19.4)	0 (0.0)
Anemia	7 (11.3)	1 (1.6)	2 (6.5)	1 (3.2)
Blood alkaline phosphatase increased	7 (11.3)	1 (1.6)	0 (0.0)	0 (0.0)
Hypothyroidism	7 (11.3)	0 (0.0)	8 (25.8)	1 (3.2)
Fatigue	6 (9.7)	0 (0.0)	0 (0.0)	0 (0.0)
Gamma-glutamyltransferase increased	6 (9.7)	1 (1.6)	1 (3.2)	0 (0.0)
White blood cell count decreased	6 (9.7)	0 (0.0)	3 (9.7)	0 (0.0)
Blood lactate dehydrogenase increased	4 (6.5)	0 (0.0)	1 (3.2)	0 (0.0)
Decreased appetite	4 (6.5)	0 (0.0)	1 (3.2)	0 (0.0)
Diarrhea	4 (6.5)	0 (0.0)	3 (9.7)	0 (0.0)
Pyrexia	4 (6.5)	0 (0.0)	2 (6.5)	0 (0.0)

AEs were classified based on MedDRA version 26.0. AEs were graded for severity using CTCAE v5.0. Patients with multiple events for a given Preferred Term were counted once at the Preferred Term level. Events were sorted by decreasing frequency of Preferred Term in 'Any Grade' of Arm A. Events were cut per Preferred Term ≥5% of 'Any Grade' of Arm A.

Abbreviations: AE, adverse event; CTCAE v5.0, Common Terminology Criteria for Adverse Events version 5.0; TEAE, treatment-emergent adverse event.

Supplementary Table S4. Tislelizumab/ociperlimab-related TEAEs in ≥5% of patients (safety analysis set)

Preferred Term	Arm A<br>Ociperlimab + tislelizumab + BAT1706<br>(N = 62)<br>n (%)		Arm B<br>Tislelizumab + BAT1706<br>(N = 31)<br>n (%)	
	Any grade	Grade ≥3	Any grade	Grade ≥3
Patients with any tislelizumab/ ociperlimab-related TEAE	55 (88.7)	29 (46.8)	24 (77.4)	6 (19.4)
Aspartate aminotransferase increased	20 (32.3)	2 (3.2)	5 (16.1)	0 (0.0)
Alanine aminotransferase increased	19 (30.6)	1 (1.6)	5 (16.1)	0 (0.0)
Platelet count decreased	15 (24.2)	0 (0.0)	6 (19.4)	3 (9.7)
Rash	13 (21.0)	6 (9.7)	5 (16.1)	0 (0.0)
Proteinuria	13 (21.0)	5 (8.1)	10 (32.3)	1 (3.2)
Pruritus	9 (14.5)	0 (0.0)	2 (6.5)	0 (0.0)
Blood bilirubin increased	8 (12.9)	1 (1.6)	5 (16.1)	0 (0.0)
Hypertension	8 (12.9)	5 (8.1)	5 (16.1)	2 (6.5)
Hypothyroidism	7 (11.3)	0 (0.0)	8 (25.8)	1 (3.2)
Anemia	7 (11.3)	1 (1.6)	2 (6.5)	1 (3.2)
Blood alkaline phosphatase increased	7 (11.3)	1 (1.6)	0 (0.0)	0 (0.0)
Gamma-glutamyltransferase increased	6 (9.7)	1 (1.6)	1 (3.2)	0 (0.0)
Fatigue	6 (9.7)	0 (0.0)	0 (0.0)	0 (0.0)
White blood cell count decreased	5 (8.1)	0 (0.0)	3 (9.7)	0 (0.0)
Blood lactate dehydrogenase increased	4 (6.5)	0 (0.0)	1 (3.2)	0 (0.0)
Pyrexia	4 (6.5)	0 (0.0)	2 (6.5)	0 (0.0)
Decreased appetite	4 (6.5)	0 (0.0)	1 (3.2)	0 (0.0)

AEs were classified based on MedDRA version 26.0. AEs were graded for severity using CTCAE v5.0. Patients with multiple events for a given Preferred Term were counted once at the Preferred Term level. Events were sorted by decreasing frequency of Preferred Term in 'Any Grade' of Arm A. Events were cut per Preferred Term ≥5% of 'Any Grade' of Arm A.

Abbreviations: AE, adverse event; CTCAE v5.0, Common Terminology Criteria for Adverse Events version 5.0; TEAE, treatment-emergent adverse event.

Supplementary Table S5. Immune-mediated AEs by category (safety analysis set)

Category	Arm A Ociperlimab + tislelizumab + BAT1706 (N = 62) n (%)		Arm B Tislelizumab + BAT1706 (N = 31) n (%)	
	**Any grade**	**Grade ≥3**	**Any grade**	**Grade ≥3**
Patients with any immune-mediated AE	31 (50.0)	14 (22.6)	14 (45.2)	3 (9.7)
Immune-mediated pneumonitis	4 (6.5)	2 (3.2)	0 (0.0)	0 (0.0)
Immune-mediated hepatitis	2 (3.2)	2 (3.2)	2 (6.5)	1 (3.2)
Immune-mediated skin adverse reaction	23 (37.1)	7 (11.3)	7 (22.6)	1 (3.2)
Immune-mediated endocrinopathies (hypothyroidism)	7 (11.3)	0 (0.0)	8 (25.8)	1 (3.2)
Immune-mediated endocrinopathies (hyperthyroidism)	2 (3.2)	0 (0.0)	1 (3.2)	0 (0.0)
Immune-mediated endocrinopathies (thyroiditis)	1 (1.6)	1 (1.6)	1 (3.2)	0 (0.0)
Immune-mediated endocrinopathies (adrenal insufficiency)	2 (3.2)	1 (1.6)	0 (0.0)	0 (0.0)
Immune-mediated endocrinopathies (diabetes mellitus)	1 (1.6)	1 (1.6)	0 (0.0)	0 (0.0)
Other immune-mediated reactions (pancreatitis)	1 (1.6)	1 (1.6)	0 (0.0)	0 (0.0)
Other immune-mediated reactions (musculoskeletal)	1 (1.6)	0 (0.0)	0 (0.0)	0 (0.0)

AEs were graded for severity using CTCAE v5.0. Patients with multiple events within a category were counted once at the category level. imAE categories are ordered by a fixed order.

Abbreviations: AE, adverse event; CTCAE v5.0, Common Terminology Criteria for Adverse Events version 5.0.

Supplementary Table S6. Overview of immune-mediated AEs (safety analysis set)

	**Arm A**<br>**Ociperlimab + tislelizumab + BAT1706**<br>**(N = 62)**<br>**n (%)**	**Arm B**<br>**Tislelizumab + BAT1706**<br>**(N = 31)**<br>**n (%)**
Patients with any immune-mediated AE	31 (50.0)	14 (45.2)
Grade ≥3	14 (22.6)	3 (9.7)
Serious	9 (14.5)	1 (3.2)
Leading to death*	1 (1.6)	0 (0.0)
Leading to treatment discontinuation	6 (9.7)	1 (3.2)

AEs were graded for severity using CTCAE v5.0.

*Immune-mediated AEs that led to death included immune-mediated hepatitis.

Abbreviations: AE, adverse event; CTCAE v5.0, Common Terminology Criteria for Adverse Events version 5.0.

FIGURES

Supplementary Figure S1. Forest plot for ORR assessed by the investigator (ITT analysis set).

Subgroup	Responder/total (%) Arm A	Responder/total (%) Arm B	Risk difference % (95% CI)
**Overall**	23/62 (37.1)	13/32 (40.6)	−3.5 (−24.4, 17.3)
**Age group**			
<65 years	15/45 (33.3)	10/21 (47.6)	−14.3 (−39.7, 11.1)
≥65 years	8/17 (47.1)	3/11 (27.3)	19.8 (−15.6, 55.2)
**Sex**			
Male	23/57 (40.4)	12/30 (40.0)	0.4 (−21.3, 22.0)
Female	0/5 (0.0)	1/2 (50.0)	−50.0 (−100.0, 19.3)
**ECOG PS**			
0	15/39 (38.5)	7/18 (38.9)	−0.4 (−27.6, 26.8)
1	8/23 (34.8)	6/14 (42.9)	−8.1 (−40.5, 24.3)
**PD-L1 expression**			
<1%	9/33 (27.3)	8/18 (44.4)	−17.2 (−44.7, 10.4)
≥1%	14/29 (48.3)	5/14 (35.7)	12.6 (−18.4, 43.6)
**MVI status**			
Present	4/13 (30.8)	5/12 (41.7)	−10.9 (−48.4, 26.6)
Absent	19/49 (38.8)	8/20 (40.0)	−1.2 (−26.7, 24.2)
**EHS status**			
Present	10/31 (32.3)	9/19 (47.4)	−15.1 (−42.9, 12.7)
Absent	13/31 (41.9)	4/13 (30.8)	11.2 (−19.3, 41.7)
**MVI/EHS status**			
Present	13/41 (31.7)	10/21 (47.6)	−15.9 (−41.6, 9.8)
Absent	10/21 (47.6)	3/11 (27.3)	20.3 (−13.5, 54.2)
**Viral status**			
HBV	18/53 (34.0)	8/24 (33.3)	0.6 (−22.1, 23.4)
HCV	2/2 (100.0)	2/3 (66.7)	33.3 (−20.0, 86.7)
Uninfected	3/7 (42.9)	3/5 (60.0)	−17.1 (−73.6, 39.3)
**BCLC stage at study entry**			
Stage B	9/17 (52.9)	3/9 (33.3)	19.6 (−19.3, 58.5)
Stage C	14/45 (31.1)	10/23 (43.5)	−12.4 (−36.7, 12.0)
**TIGIT expression**			
<1%	9/28 (32.1)	7/16 (43.8)	−11.6 (−41.4, 18.2)
≥1%	12/32 (37.5)	6/16 (37.5)	0.0 (−29.1, 29.1)
**Alpha-fetoprotein at baseline**			
≤200 ng/mL	14/35 (40.0)	10/19 (52.6)	−12.6 (−40.3, 15.1)
>200 ng/mL	6/22 (27.3)	3/9 (33.3)	−6.1 (−42.0, 29.9)
Missing	3/5 (60.0)	0/4 (0.0)	60.0 (17.1, 100.0)
**Alpha-fetoprotein at baseline**			
≤400 ng/mL	14/39 (35.9)	10/19 (52.6)	−16.7 (−43.8, 10.3)
>400 ng/mL	6/18 (33.3)	3/9 (33.3)	0.0 (−37.7, 37.7)
Missing	3/5 (60.0)	0/4 (0.0)	60.0 (17.1, 100.0)

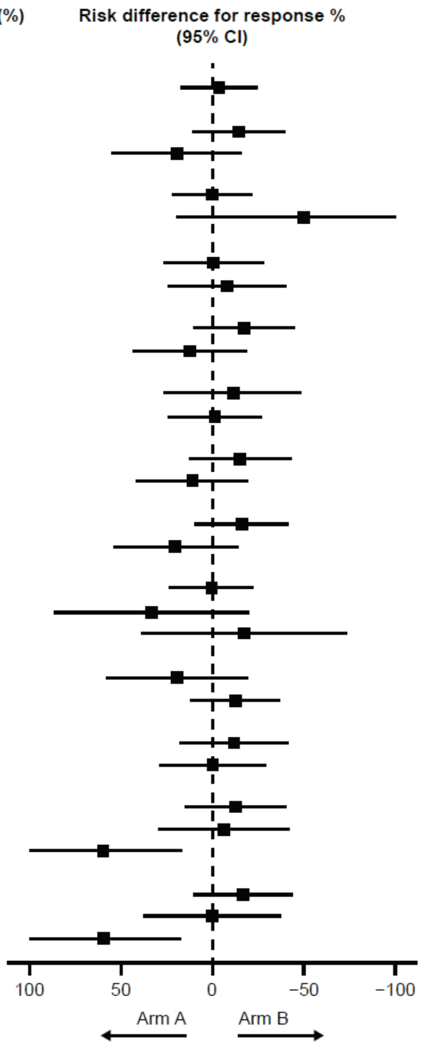

Risk difference was estimated along with its 95% CIs constructed by a normal approximation and Sato's variance estimator.

One HBV and HCV co-infected patient was counted under the HBV category only. Two patients in Arm A were excluded from the TIGIT expression subgroup analysis as they have no result of TIGIT expression due to technical issues.

Abbreviations: BCLC, Barcelona Clinical Liver Cancer; CI, confidence interval; ECOG, Eastern Cooperative Oncology Group; EHS, extrahepatic spread; HBV, hepatitis B virus; HCV, hepatitis C virus; ITT, intention-to-treat; MVI, macrovascular invasion; ORR, objective response rate; PD-L1, programmed death protein-ligand 1; TIGIT, T-cell immunoglobulin and immunoreceptor tyrosine-based inhibitory motif domain.

Supplementary Figure S2. Forest plot for PFS assessed by the investigator (ITT analysis set)

Subgroup	Event/total Arm A	Event/total Arm B	HR (95% CI)
**Overall**	46/62	22/32	1.10 (0.66, 1.84)
**Age group**			
<65 years	33/45	12/21	1.39 (0.72, 2.70)
≥65 years	13/17	10/11	0.69 (0.30, 1.61)
**Sex**			
Male	41/57	20/30	1.09 (0.64, 1.86)
Female	5/5	2/2	1.08 (0.19, 6.05)
**ECOG PS**			
0	29/39	13/18	0.90 (0.46, 1.73)
1	17/23	9/14	1.43 (0.63, 3.23)
**PD-L1 expression**			
<1%	25/33	13/18	1.18 (0.60, 2.32)
≥1%	21/29	9/14	1.09 (0.50, 2.38)
**MVI status**			
Present	11/13	4/12	2.08 (0.65, 6.62)
Absent	35/49	18/20	0.84 (0.47, 1.49)
**EHS status**			
Present	21/31	11/19	1.37 (0.66, 2.85)
Absent	25/31	11/13	0.74 (0.36, 1.52)
**MVI/EHS status**			
Present	30/41	11/21	1.64 (0.82, 3.28)
Absent	16/21	11/11	0.55 (0.25, 1.21)
**Viral status**			
HBV	42/53	18/24	1.03 (0.59, 1.80)
HCV	0/2	2/3	0.00 (0.00, NE)
Uninfected	4/7	2/5	1.02 (0.17, 6.13)
**BCLC stage at study entry**			
Stage B	13/17	9/9	0.52 (0.22, 1.23)
Stage C	33/45	13/23	1.51 (0.79, 2.87)
**TIGIT expression**			
<1%	21/28	11/16	1.66 (0.76, 3.59)
≥1%	25/32	11/16	0.99 (0.48, 2.01)
**Alpha-fetoprotein at baseline**			
≤200 ng/mL	24/35	12/19	1.30 (0.65, 2.61)
>200 ng/mL	19/22	7/9	1.15 (0.48, 2.76)
Missing	3/5	3/4	0.00 (0.00, NE)
**Alpha-fetoprotein at baseline**			
≤400 ng/mL	28/39	12/19	1.43 (0.72, 2.82)
>400 ng/mL	15/18	7/9	1.01 (0.41, 2.51)
Missing	3/5	3/4	0.00 (0.00, NE)

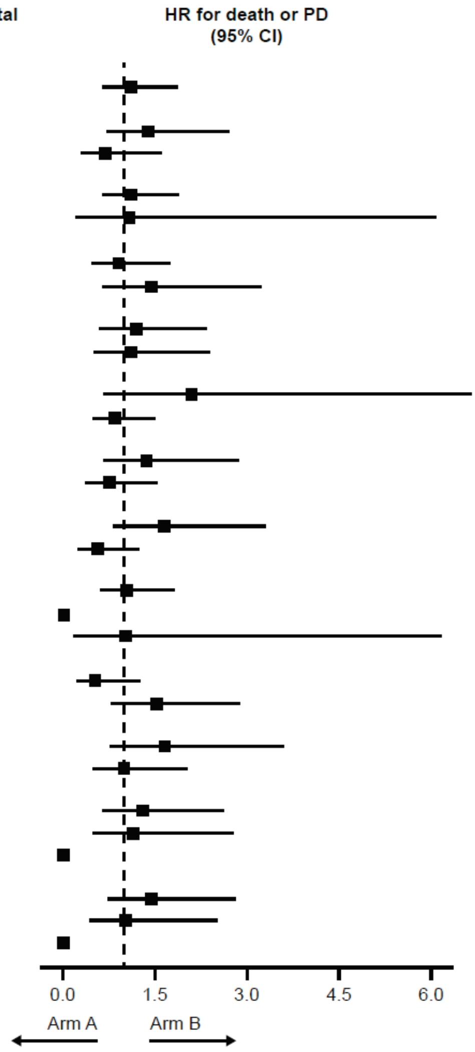

HR and its 95% CIs were estimated using an unstratified Cox regression model. Efron method was used to handle ties if there were any.

One HBV and HCV co-infected patient was counted under the HBV category only. Two patients in Arm A were excluded from the TIGIT expression subgroup analysis as they have no result of TIGIT expression due to technical issues.

Abbreviations: BCLC, Barcelona Clinical Liver Cancer; CI, confidence interval; ECOG, Eastern Cooperative Oncology Group; EHS, extrahepatic spread; HBV, hepatitis B virus; HCV, hepatitis C virus; HR, hazard ratio; intention-to-treat; MVI, macrovascular invasion; NE, not estimable; PD, progressive disease; PD-L1, programmed death protein-ligand 1; PFS, progression-free survival; TIGIT, T-cell immunoglobulin and immunoreceptor tyrosine-based inhibitory motif domain.

Supplementary Figure S3. Forest plot for OS (ITT analysis set).

Subgroup	Event/total Arm A	Event/total Arm B	HR (95% CI)
**Overall**	28/62	14/32	1.01 (0.53, 1.92)
**Age group**			
<65 years	19/45	8/21	0.99 (0.43, 2.26)
≥65 years	9/17	6/11	1.10 (0.38, 3.13)
**Sex**			
Male	25/57	13/30	0.97 (0.50, 1.90)
Female	3/5	1/2	1.54 (0.16, 14.87)
**ECOG PS**			
0	16/39	8/18	0.83 (0.36, 1.95)
1	12/23	6/14	1.36 (0.51, 3.65)
**PD-L1 expression**			
<1%	14/33	8/18	0.93 (0.39, 2.21)
≥1%	14/29	6/14	1.04 (0.40, 2.72)
**MVI status**			
Present	9/13	5/12	1.59 (0.53, 4.79)
Absent	19/49	9/20	0.91 (0.41, 2.01)
**EHS status**			
Present	13/31	5/19	1.76 (0.63, 4.95)
Absent	15/31	9/13	0.54 (0.24, 1.24)
**MVI/EHS status**			
Present	20/41	7/21	1.53 (0.64, 3.61)
Absent	8/21	7/11	0.49 (0.18, 1.36)
**Viral status**			
HBV	24/53	10/24	1.02 (0.49, 2.13)
HCV	0/2	2/3	0.00 (0.00, NE)
Uninfected	4/7	2/5	1.56 (0.28, 8.60)
**BCLC stage at study entry**			
Stage B	5/17	7/9	0.13 (0.03, 0.54)
Stage C	23/45	7/23	1.98 (0.85, 4.62)
**TIGIT expression**			
<1%	15/28	7/16	1.50 (0.61, 3.71)
≥1%	13/32	7/16	0.79 (0.31, 1.98)
**Alpha-fetoprotein at baseline**			
≤200 ng/mL	13/35	6/19	1.46 (0.55, 3.86)
>200 ng/mL	14/22	5/9	1.16 (0.42, 3.21)
Missing	1/5	3/4	0.00 (0.00, NE)
**Alpha-fetoprotein at baseline**			
≤400 ng/mL	16/39	6/19	1.64 (0.64, 4.23)
>400 ng/mL	11/18	5/9	1.05 (0.36, 3.03)
Missing	1/5	3/4	0.00 (0.00, NE)

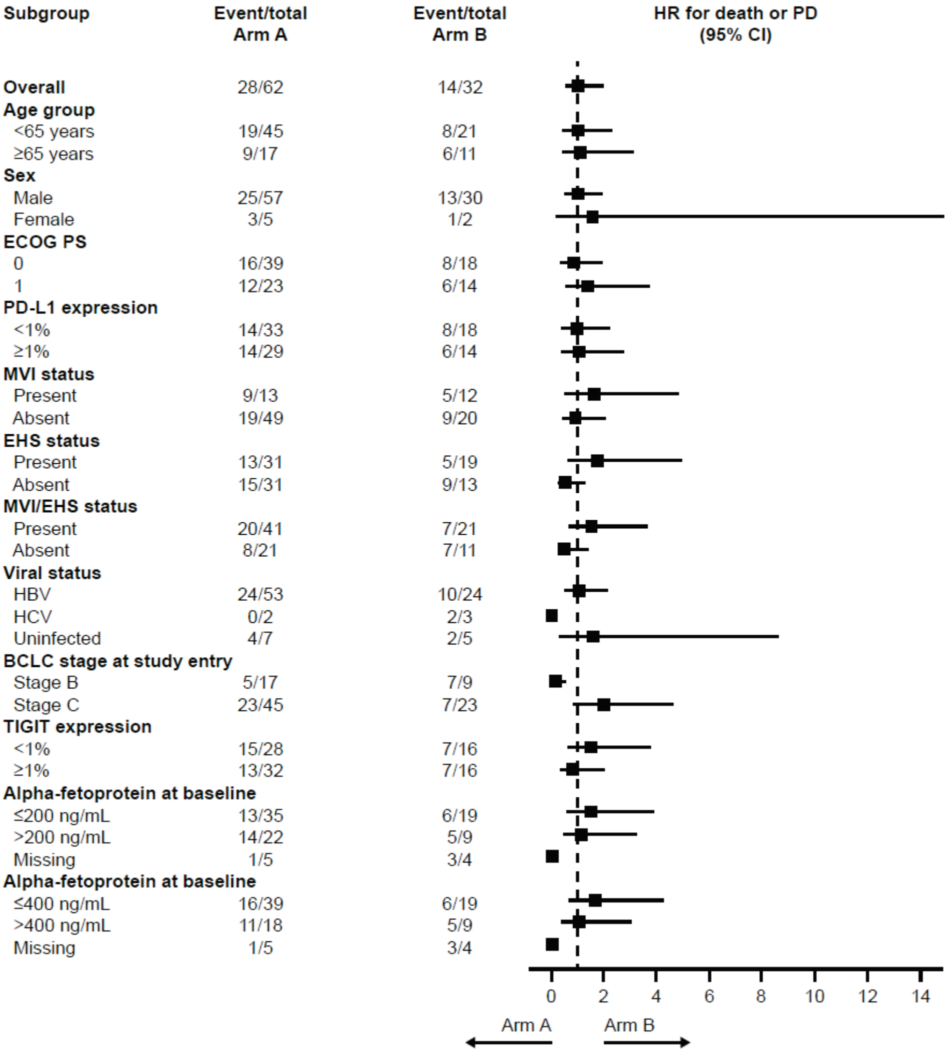

HR and its 95% CIs were estimated using an unstratified Cox regression model. Efron method was used to handle ties if there were any.

One HBV are HCV co-infected patient was counted under the HBV category only. Two patients in Arm A were excluded from the TIGIT expression subgroup analysis as they have no result of TIGIT expression due to technical issues.

Abbreviations: BCLC, Barcelona Clinical Liver Cancer; CI, confidence interval; ECOG, Eastern Cooperative Oncology Group; EHS, extrahepatic spread; HBV, hepatitis B virus; HCV, hepatitis C virus; HR, hazard ratio; ITT, intention-to-treat; MVI, macrovascular invasion; NE, not estimable; OS, overall survival; PD, progressive disease; PD-L1, programmed death protein-ligand 1; TIGIT, T-cell immunoglobulin and immunoreceptor tyrosine-based inhibitory motif domain.
